# Chemotherapeutic potential of hesperetin for cancer treatment, with mechanistic insights: A comprehensive review

**DOI:** 10.1016/j.heliyon.2022.e08815

**Published:** 2022-01-23

**Authors:** Md Sohel, Habiba Sultana, Tayeba Sultana, Md. Al Amin, Suraiya Aktar, Md. Chayan Ali, Zahed Bin Rahim, Md. Arju Hossain, Abdullah Al Mamun, Mohammad Nurul Amin, Raju Dash

**Affiliations:** a Department of Biochemistry and Molecular Biology, Mawlana Bhashani Science and Technology University, Santosh, Tangail 1902, Bangladesh; b Department of Biotechnology and Genetic Engineering, Mawlana Bhashani Science and Technology University, Santosh, Tangail 1902, Bangladesh; c Department of Biochemistry and Molecular Biology, Rajshahi University, Rajshahi, Bangladesh; d Department of Biotechnology and Genetic Engineering, Islamic University, Kushtia 7003, Bangladesh; e Department of Pharmacy, BGC Trust University Bangladesh, Chittagong 4381, Bangladesh; f Department of Pharmacy, Atish Dipankar University of Science and Technology, Dhaka 1230, Bangladesh; g Pratyasha Health Biomedical Research Center, Dhaka 1230 Bangladesh; h Department of Anatomy, Dongguk University College of Medicine, Gyeongju 38066, Republic of Korea

**Keywords:** Cancer, Hesperetin, Anticancer agents, ADME/Tox

## Abstract

**Background:**

Cancer has become a significant concern in the medical sector with increasing disease complexity. Although some available conventional treatments are still a blessing for cancer patients, short-and long-term adverse effects and poor efficiency make it more difficult to treat cancer patients, demonstrating the need for new potent and selective anticancer drugs. In search of potent anticancer agents, naturally occurring compounds have always been admired due to their structural diversity, where Hesperetin (HSP) may be one of the potent candidates.

**Purpose:**

We aimed to summarize all sources, pharmacological properties, anticancer activities of HSP against numerous cancers types through targeting multiple pathological processes, mechanism of HSP on sensitizing the current anti-cancer agents and other phytochemicals, overcoming resistance pattern and determining absorption, distribution, metabolism, excretion, and toxicity (ADME/Tox).

**Methods:**

Information was retrieved from PubMed, Science Direct, and Google Scholar based on some key points like Hesperetin, cancer name, anticancer resistance, nanoformulation, and ADME/Tox was determined by *in silico* approaches.

**Result:**

HSP is a phytoestrogen present in citrus fruits in a high concentration (several hundred mg/kg) and exhibited anti-cancer activities through interfering at several pathways. HSP can suppress tumor formation by targeting several cellular proteins such as cell cycle regulatory, apoptosis, metastatic, tyrosine kinase, growth factor receptor, estrogen metabolism, and antioxidant-related protein.HSP has shown remarkable synergistic properties in combination therapy and has been reported to overcome multidrug cancer resistance drugs, leading to an improved defensive mechanism. These anticancer activities of HSP may be due to proper structural chemistry.

**Conclusion:**

Overall, HSP showed potential anticancer activities against all cancer and possess better pharmacokinetic properties. So this phytochemical alone or combination with other agents can be an effective alternative drug for cancer treatment.

## Highlights

• Hesperetin is potential natural compound for its attributes in various anticancer activities.
• Hesperetin can modulate diverse signaling pathways in cancer cells related to growth, metastasis, and apoptosis.
• Hesperetin also increases chemosensitivity in chemotherapy in a synergistic approach.
• Hesperetin processes less toxicity in human body but more bioavailability, conferring its application in clinical settings.

## Graphical abstract

**Figure:**
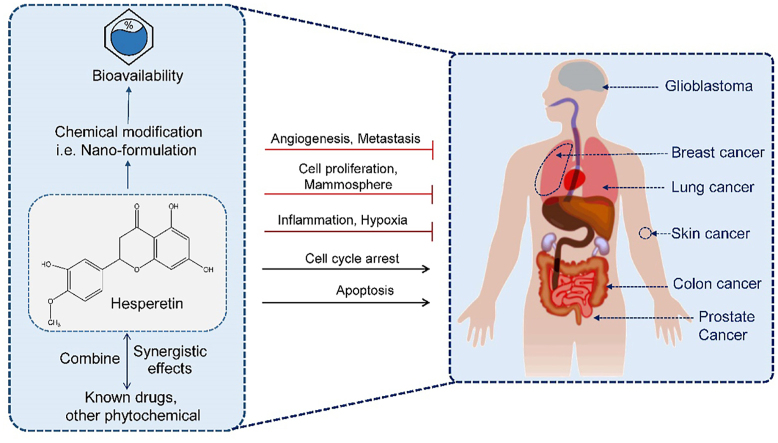

## 1 Introduction

Around the world, cancer has become a significant public health problem due to higher incidence and mortality. The new case of cancer patients is increasing every day, particularly in societies with low and medium-income [1]. According to the Global Cancer Statistics 2020; GLOBOCAN, a total of 19,292,789 patients were affected, and 9,958,133 patients have died from cancer [1]. The list of common cancer among the patients have included breast cancer, which already bypassed lung cancer with around 2.3 million new incidences (11.7%), followed by lung (11.4%), prostate (7.3%), and stomach (5.6%) cancers. In the case of mortality, maximum patients died by lung cancer(18%), followed by colorectal (9.4%), liver(8.3%), and breast cancer (6.9%) cancers [1]. Such high cases of death are due to a lack of overall knowledge about cancer and proper therapeutics. The invention of a new drug is quite challenging, time-consuming, and on the other side, due to the side effects of the existing conventional treatment such as chemotherapy [2] and radiotherapy [3], increasing the burden more and making it more challenging to treat the patients of cancer. However, introducing new plant-based compounds from natural origin may consider a new and trustworthy curative component for treating different numerous disease like infectious [4] to noninfection cancers [5] in humans based on their selective molecular targets. Natural products and dietary supplements have grown in popularity in the clinical setting throughout the years. Natural sources i,e plants, animals, and microorganisms, serve as about 75% of the clinically used anti-cancer drugs [6] and have been used medicinally for decades, usually in complementary and alternative medicine [7].

HSP is a phytoestrogen, naturally occurring flavone found in the fruit peel of *Citrus aurantium L*. (Rutaceae) and has recently gained attention for its anti-tumour properties [8]. HSP exhibits a cytotoxic mechanism towards multiple cancer cells such as breast cancer [9], pancreatic Cancer [10], prostate cancer [11], glioblastoma [12], liver cancer [13], kidney cancer [14], colon cancer [15], lung cancer [16], oral cancer [17], esophageal cancer [18], osteosarcoma [19], ovarian cancer [20], thyroid [21], leukemia [22] and some other cancers showing that HSP could be a promising cancer treatment candidate. The use of structure-activity relationship (SAR) studies of HSP can predict several biological activities like antioxidant and anticancer. In carcinogenesis, HSP can halt cancer initiation and progression by regulating multiple cellular mechanisms such as arresting the cell cycle, cell proliferation, metastasis, angiogenesis, epigenetic factors, and apoptosis-related cell death. Moreover, HSP may serve as a synergistic drug with some chemotherapy regimens for the treatment of cancer patients. The major advantage of using HSP cancer treatment is its availability in nature, structural chemistry, formation of nano-carrier to increase bioavailability, pharmacokinetic profile, and less toxicity. In previous studies, HSP provided a wide range of medicinal and therapeutic quality against numerous other diseases, but the extensive molecular mechanism evaluations of anticancer effects of HSP are still rare. Therefore, we aimed to review and summarise existing scientific reports on the anti-cancer effects of HSP.

## 2 Methodology

The information about HSP i,e sources, chemistry, structure-activity relationship, nanoformulation, anticancer activities, synergistic effect with other phytochemicals and known drug and toxicity was conducted by considering all scientific published articles until June 30, 2021, through several searching databases like PubMed, Science Direct, and Google Scholar. Some information was also retrieved from the textbook. In the case of searching, the English language system was considered. HSP, cancer types, anti-cancer mechanism, anticancer resistance, nanoformulation, synergistic activities, and toxicity were used as the main keywords during the searching time. Information that is irrelevant to our study was excluded. In-silico approaches conducted pharmacokinetics or ADME/Tox prediction through computational tools such as Schrodinger's QuickPro modules [23] and online accessible server admetSAR [24], and SwissADME [25] were used.

## 3 Sources

HSP is a natural flavonoid containing various pharmacological properties, mainly found in the fruit of citrus species, including- oranges, grapefruit, and tangerines [26]. It is found mainly in *Citrus aurantinum*, *Citrus sinensis,* and *Citrus limon* [27]. Various fruit juices, such as orange and grapefruit juice, contain a significant amount of HSP [28, 29]. It has been found that the fermented orange juice includes an increased amount of HSP than the original orange juice [30]. HSP has also been extracted from the callus culture of *Citrus aurantifolia* at a large scale [31]. Peel of citrus fruits and mandarin contain a high level of HSP. It has been described that orange and mandarin are good sources of HSP [32]. Again, it has been identified in citrus honey [33]. In addition to these, HSP has also been isolated from the *Cordia sebestena* flower extract using an antioxidant assay-guided technique [34].In addition to mentioned sources, lemons are a rich source of HSP [35]. HSP is also found in the *Fructus aurantii* extract, which is the mature and dried fruit of *Citrus aurantinum* [36]. Hesperidin can be a good source of HSP as it can be produced from the hydrolysis of hesperidin [37]. Usually, half of orange and half of mandarin contains about 130 mg of HSP [1], where the average intake of HSP has been estimated to be around 28.3 mg/d [38]. This phytochemical can be isolated using numerous solvent acetone [34], through various spectroscopic techniques [34]. Furthermore, the isolation of hesperetin can possible with antopxidant assay guided [34].

## 4 Chemistry

**Figure 1:**
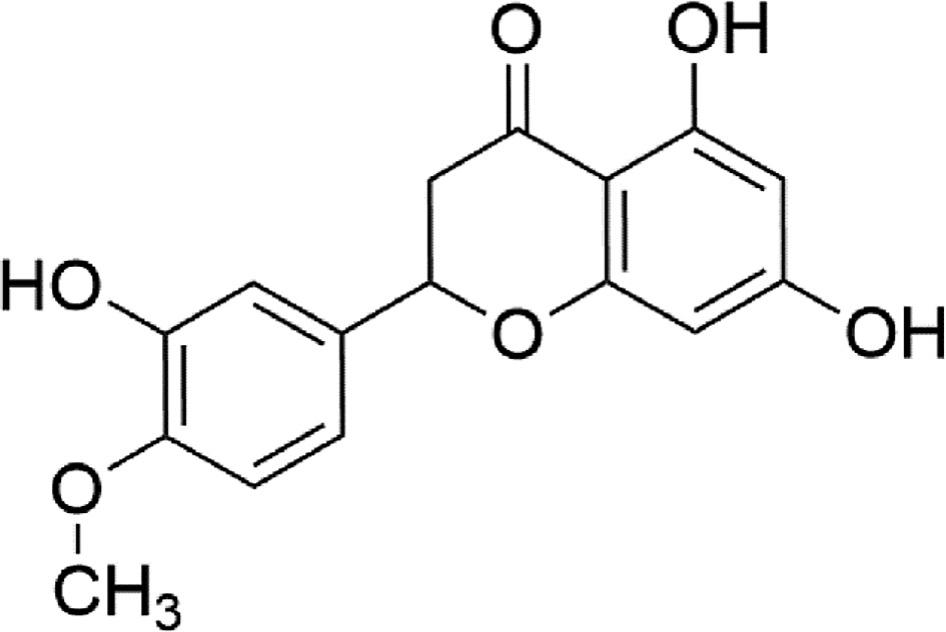
Chemical structure of Hesperetin.

HSP is the 4′-methoxy derivative of eriodictyol, a flavanone. The chemical formula of HSP is C_16_H_14_O_6,_ where the IUPAC name of this phytoestrogen is 5,7-dihydroxy -2-(3-hydroxy-4-methoxyphebyl)-2,3--dihydrochromen-4-one. It contains 22 heavy atom counts, 1 covalently-bonded unit amount, the topological polar surface area is 96.2 Å^2^, and no formal charge. The molecular weight of HSP is 302.28 g/mol, but the exact mass is 302.079 [39]. HSP is aglycone based on hesperidin, where hesperidin contains an aglycone, HSP (methyl eriodictyol) which is bonded to rutinose [40]. HSP can be produced with combined alkaline hydrolysis between phloroglucinol and HSP acid [41]. Three hydroxyl groups in the heterocyclic and aromatic ring are available and responsible for several biological activities [40]. Figure 1. Portrays the chemical structure of HSP.

## 5 Structure-activity relationship (SAR)

Flavonoids and flavanones contain hydroxylated phenolic structures and are attributed to several biological activities, most of which depend on the arrangement of functional groups around their core structures [42]. The HSP is a potent flavanone and has strong health-stimulating effects primarily due to polyphenolic structures [43]. The overall amount and arrangement of functional hydroxyl groups significantly impact antioxidant activity, reflecting its free radical scavenging or metal chelating activities [44]. HSP contains three hydroxyls at the C-3, C-5, and C-7, respectively, carbonyl group (the 4(=O) on the C ring with a double bond of C5–C6 at the A ring. But compared to other phytochemicals, HSP has double bond deficiency at C2 = C3, resulting in decreased antioxidant activity for HSP [45]. In contrast, in the aglycone form, HSP's antioxidant activity is unaffected by the 5′,7′-di-hydroxy substitution, with its three OH substitutions and lack of a 5′,7′-di-hydroxy structure [44]. HSP has a topological polar surface area (TPSA) is about 96.2 Å^2^. Si-Ahmed et al. summarised that proper TPSA is associated with the anticancer-activity of alkaloids by inhibiting telomerase and aromatase enzymes [46]. HSP has two enantiomers are namely R- and S-HSP [47]. This type of enantiomers in flavones, i,e (S) and (R) naringenin, could downregulate miR-17-3p expression in human colon adenocarcinoma [48]. HSP-metal complex, i,e HST-Cu (II) attributed better biological activities than free HSP, including cytotoxicity, antioxidant, autophagy, and apoptosis. The coordination of Cu between 4 and 5 positions of the condensed ring structure may be linked to improveds antioxidant activity, increasing its activities of stabilizing unpaired electrons in the time of free radical scavenging in human A549 lung adenocarcinoma cells [49].

## 6 Nano formulation strategies of HSP with aiming better bioavailability

*Citrus* fruits contain HSP, a naturally occurring plant bioflavonoid with anti-inflammatory and anti-carcinogenic properties. However, HSP's low aqueous solubility restricts its application. Before using HSP in a clinical trial, its solubility in an aqueous solution and tumor-specific accumulation needs to be improved. The use of HSP in conjunction with nanomaterials is a novel approach to treating cancer cells that increase the bioavailability of HSP. In this regard, HSP conjugated with gold nanoparticles (Au-mPEG(_5000_)-S-HP NPs), *Chitosan folate*, and Polyethylene glycolated (PLGA) carrier are widely used to increase anti-cancer activity. Gurushankar et al. summarized that nanoparticles from HSP increased particle size in the range from 55 to 180 nm and increased anti-cancer activities by generating ROS, damaging DNA, and apoptotic markers in HETNPs treated cells in only HSP treatment [17]. *Chitosan folate* hesperetin (CFH) nanoparticle inhibited colony formation, cell proliferation, and induced apoptosis by regulating proapoptotic gene expression with the IC50 value of 28 μM in colorectal cancer cells [50]. The anti-cancer activity of HSP-loaded PLGA nanoparticle systems was considerable through increasing apoptosis, oxidative stress, decreasing lipid peroxidation in C6 glioma cell line at a time, and dose-dependent manner [51]. Krishnan reported that Au-mPEG(_5000_)-S-HP NPs increased anti-cancer effect through cell inflammation-mediated decreasing mast cell count, TNF-α and NF-κB, glycoconjugates in hepatocellular carcinogenesis [52]. Anti-androgen medication bicalutamide (BCT) and HSP co-delivery in chitosan (CS) coated polycaprolactone (PCL) nanoparticles (NPs) enhanced the kinetic solubility of BCT and HSP around 61.66 and 6.75 times, respectively with increasing cytotoxicity, cell cycle arrest apoptosis [53]. Sheokand et al. summarised that nanocrystalline solid dispersions (NSD) of HSP increased bioavailability by increasing absorption rate and 4-fold reduction Tmax in DMBA induced breast cancer in Sprague-Dawley (SD) rat [54]**.** These findings suggest that HSP can be integrated into solid nanoparticles that make them more stable to improve biological functions.

## 7 Anti-cancer perspectives of HSP

**Figure 2:**
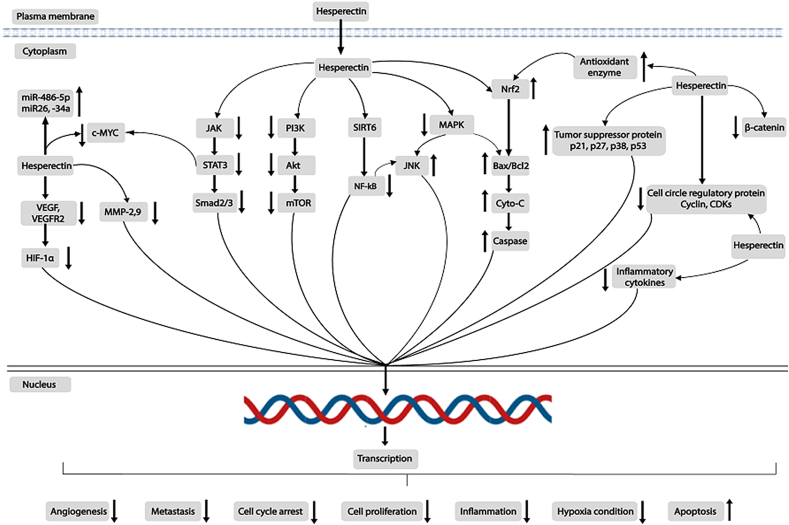
A comprehensive schematic diagram about the modulatory effects of Hesperetin on various cell signaling pathways against multiple cancer to regulate cancer management.

Previously, *in vitro and in vivo* studies on HSP have shown promising anticancer activities against numerous cancer through targeting multiple pathways. More specifically, HSP could regulate some regulatory proteins like cell cycle, apoptosis, metastatic, tyrosine kinase, growth factor receptor, estrogen metabolism, and related proteins (Figure 2).

### 7.1 Breast cancer

Breast cancer is a common cause of mortality in women as it is a metastatic disease that frequently spreads to other organs, e.g., the bone, liver, brain, and lung. The fundamental factors of breast cancer development include sex, estrogen, aging, genetic mutation, family history, and lifestyle; however, the survival rate is higher if the disease is detected early in life [55]. Numerous studies have proved that HSP is an effective natural flavonoid successfully used for breast cancer treatment. HSP can suppress cancer cell proliferation, viability, migration, invasion, mammosphere, and colony formation and stimulate DNA damage and apoptosis. Being a promising anticancer agent, HSP (100 μM) showed cytotoxicity on cells and prevented mammosphere, colony formation, and migration through elevating the mRNA level of p53, NOTCH1, PPARG, and reduced β-catenin resulted in apoptosis with cell cycle arrest G0/G1 phase in MCF-7 breast cancer cells [56]. Another study on a similar cell line by Choi showed that HSP (1–100 μM) administration induces apoptosis and inhibits cell proliferation after 48–72 h treatment [57]. HSP decreased CDK 2, CDK 4, and cyclin E, cyclin D expression when administered at the 50–100 μM concentration for 48–72 h. Additionally, HSP could raise p21Cip1 and p27Kip1 expression, CDK4-p21Cip1 complex formation, and arrest cell cycle at the G1 phase [57]. Like other mechanisms, HSP could regulate some cancer-associated enzymes in breast cancer. A recent study suggests that aryl hydrocarbon receptor (Ahr) is blocked, and CYP1A1, 1A2, 1B1 expression is down-regulated when treated with HSP (1–20 μM) in MCF-7 breast cancer cells [58]. Additionally, HSP suppressed aromatase enzyme activity, cyclin D1, CDK4, Bcl-xL, pS2 and induces CCAAT/C/EBP, pERK-1&-2, p57Kip2 expression, which contributes to reducing the tumour growth in MCF-7 breast cancer cells and female athymic mice model both *in vivo* and *in vitro* [59, 60]*.* To see the effect of HSP on human epidermal growth factor receptor 2 (HER2), researchers performed a study on HER2 overexpressed breast cancer cell (MCF-7/HER2) and MCF-7/EV cell and observed that at 95 μM concentration, HSP decreased HER2, MMP-9, Rac1 expression, lamellipodia formation, and arrested cell cycle at G2/M phase; therefore reducing cell viability, invasion, migration and promoting apoptosis [61]. Palit *et al.* experimented with HSP (20–200μM) on MCF-7, MCF-10A, HMEC, MDA-MB 231 breast carcinoma and stated this compound is associated with increasing ROS production, release cyto-C, Bax/Bcl-2 ratio, caspase-9,-3, 7, PARP cleavage, JNK, and sk1activation, and activating ASK1/JNK pathway [62]. In MDA-MB-231 breast cancer cells, HSP reduces glucose uptake by down-regulating glucose transporter 1 (GLUT1) and 4 (GLUT4), inhibits insulin receptor-beta subunit (IR-beta) phosphorylation and Akt, leading to suppressed cell proliferation in response to 5–100 μM concentration [63]. Abdallah and his colleagues found that HSP can also increase the expression of miR-486-5P that is responsible for reducing cell viability, clonogenicity, and metastatic potential in similar cell lines [64]. According to Chandrika *et al.,* 10–500 μM of HSP causes apoptosis and reduces cell growth in MDA-MB-231 and SKBR3 breast cancer cells. Dietary flavonoid HSP inhibits HER2 Tyrosine Kinase (HER2-TK) activity, causes MMP loss, chromatin condensation, activates caspase-8 and-3, resulting in cell cycle arrest at the G2 phase, which decreases the SKBR3 in MDA-MB-231 breast cancer cell growth [65]. In 4T1 murine breast cancer cells, HSP could induce apoptosis and stop metastasis through down-regulating MMP-9 expression and arresting cell cycle at Sub G1 phase when administered at 50–100 μM concentration [66].

In summary, from the existing mechanistic of breast cancer studies, it can be understood that HSP causes upregulation of tumour suppressor gene that can control cell cycle progression; this phytoestrogen also regulates estrogen metabolism, which is the main culprit in case of breast cancer and induced both extrinsic and intrinsic pathways apoptosis leading to cell deaths. Besides these activities, HSP can suppress some tumour-related growth factors within the inhibition of metastasis.

### 7.2 Lung cancer

Global health is threatened by lung cancer, which has been ranked the most frequent cancer globally by the International Agency for Research on Cancer (11.4% of all cancer) with over new 2206771 diagnoses and 1796144 deaths worldwide in 2020 [1]. However, like other natural compounds***,*** HSP can also be used to treat lung cancer because of its capacity to stimulate several responsible targets. HSP treated in H522 lung cancer cells mediated apoptosis with initiating Fas death receptor/extrinsic pathway, resulting in upregulating Bax, caspase-3, and caspase-9 in a dose-dependent manner [16]. Similarly, HSP showed significant antiproliferative effects in H441 lung cancer cells by inhibiting transforming growth factor β and reducing glucose uptake in a cancer cell by downregulating glucose transporter expression [67]. A study of HSP and copper showed that HSP (16 μM) reduces angiogenesis through the mitochondria-mediated pathway by activating TRIAL cytotoxic protein that stimulates several mechanisms of apoptosis in lung cancer cells [49]. Interleukin (IL)-1, a pro-inflammatory cytokine, has been linked to enhanced cell proliferation, angiogenesis, adhesion, invasion, promotion, and metastasis in lung cancer through the expression of corresponding biomarker proteins. IL-1 is associated with activating inflammatory indicators such as cyclooxygenase 2(COX-2) and inducing cell proliferation through activation of mitogen-promoting kinases (COX-2) [68, 69]. By inhibiting IL-1β, HSP (100 μM) decreases COX-2 expression and its regulation in translation level in A549 lung cancer cell and PGE2 synthesis, indicating its anti-inflammatory and anti-cancer potential in human lung cancer cells [70]. Lakhsmi *et al.* revealed that HSP treatment in Swiss albino mice prevents cancer formation by alleviating LPO, modulating antioxidants enzymes like decreased NF-kB, PCNA, and CYP1A1. This analysis shows that HSP has chemopreventive potential against B[a]P-induced lung cancer due to its free radical scavenging, antioxidant, anti-inflammatory, and antiproliferative activities [71].

Overall, in lung cancer treatment HSP induced apoptosis, decreed glucose transportation, suppressed cell cycle progression, and inflammation.

### 7.3 Prostate cancer

Prostate cancer is the most prevalent cancer among males, typically diagnosed in older men over 50 [72]. Diet, physical activity, and familial inheritance are responsible for the advancement of prostate cancer [72]. The majority of prostate cancers are adenocarcinomas, which contain many similarities with other cancers, e.g., breast and colon cancer [73]. HSP plays a significant role in lowering prostate cancer risk and treating it successfully. With similar fashion in other cancers, HSP (0–100 μM, 24–48 h) triggered apoptosis on human pancreatic cell line through attenuating Bcl-xL but increasing the expression of Bax and BAD and inhibiting cell proliferation via reducing the expression of NF-κβ [74]. *In vitro* study revealed that when PC-3 cells were exposed to HSP (0–700 μM), cell proliferation and viability were reduced after 48 h treatment. In addition, HSP (450–500 μM) increased IL-6 gene expression and attribute phosphorylation i,e signal transducer and activator of transcription 3 (STAT 3), extracellular signal-regulated kinase ½ (ERK1/2) and AKT signalling pathways, therefore arresting G0/G1 phase [75]. On the other hand, Arya *et al.* explained that HSP at 12.5–200 μM concentration was associated with cell cycle arrest at the G1/S phase and increased mitochondrial membrane depolarization caused apoptosis and reduced cell viability [53]. Furthermore, Shirzad and his co-workers proved that PC-3 prostate cancer cell undergoes apoptosis and significantly reduce cell proliferation when exposed to HSP (0–1000 μM), but they did not evaluate the impact of HSP on intracellular signalling and regulatory factors [11]. HSP can stop cell proliferation, decrease cell viability, influence apoptosis, and target many signalling pathways and proteins, consequently damaging prostate cancer cells.

Largely, HSP has limited activities on preventing prostate cancer treatment, where it can induce caspase and Bax/Bcl2 mediated apoptosis and regulate some inflammatory markers.

### 7.4 Colon cancer

The third most common cause of global cancer is colon cancer [50]. Phytochemicals such as HSP, an important bioactive compound with antioxidant and anti-carcinogenic properties abundant in citrus fruits [76]. Zhang showed that the effects of HSP on hepatocellular carcinoma cells are commendable. HSP inhibits proliferation and cell viability and increases apoptosis via activating mitochondrial-mediated pathways, where ROS, ATP, Ca2+, Cyto- C, AIF, and Apaf-1 were upregulated with downregulating Bcl2 in a dose-dependent manner [77]. HSP therapy also decreased renal hemorrhage and colon polyps by lowering cyclooxygenase-2 (COX-2) and carcinoembryonic antigen levels (CEA) and oxidative stress in 1,2 dimethylhydrazine (20 mg/kg body weight/day) induced mice model [15]. On the other hand, HSP (20 mg/kg body weight/day) supplementation on DMH treated rat decreases tumour multiplicity through the upregulation of antioxidant enzymes SOD, CAT, GPx [78]. Similarly, a study conducted by *Nalini et al.* HSP (10,20,30 mg/kg body weight/day) increased antioxidant enzyme activity i,e, SOD, CAT, glutathione, and glutathione-dependent enzymes (glutathione S-transferase (GST), glutathione peroxidase (GPx), and glutathione reductase (GR) in chemically induced colonic carcinogenic cells [79]. HSP (0–100 μM) treatment decreased cell viability and triggered apoptosis by activating the c-Jun-N-Terminal kinase(JNK) pathway in human cancer cell line HCT-116 [80]. Sivagami *et al.* summarized that HSP showed an inhibitory effect on human carcinoma cell HT-29 (5–100 μM) via the mitochondrial-mediated apoptosis induction by increasing Bax, caspase3 concomitant downregulation antiapoptotic protein BCL-2 [81].

Comprehensively, HSP mediated apoptosis-related cell death by activating both extrinsic and intrinsic apoptotic proteins and regulating antioxidant enzymes in colon cancer treatment. Furthermore, it regulates major signalling pathways and growth factors associated with cancer progression.

### 7.5 Liver cancer

Liver cancer is a heterogeneous disease caused by hepatocellular carcinoma (HCC), which develops from chronic liver inflammation and fibrosis. Hepatitis B, C virus and aflatoxin infection, excessive alcohol consumption, obesity, metabolic disorders, diabetes are all responsible for liver cancer [82]. HSP, a natural flavonoid, contains the capacity to treat liver cancer by inducing apoptosis, cancer cell damage and reducing liver injury, enlargement, and fibrosis. An *in vivo* study performed by M. Miler *et al.* on male, Wistar rats, revealed that HSP promotes cancer cell death when administered orally at 15 mg/kg. Apart from this, HPS increased antioxidant enzyme activity, including superoxide dismutase (SOD), glutathione reductase (GR), catalase (CAT) [83]. Another *in vivo* study on Sprague-Dawley rats proved that HSP (50 mg/kg/day) induces apoptosis and regulate oxidative stress through upregulating Fas/FasL and caspase-8,-3 expression, albumin level, and lowered the level of hepatic glutathione (GSH), hepatic malondialdehyde (MDA), and Bcl-2 expression [13]. Kong *et al.* performed research on HSC-T6 cell and male C57 mice to estimate the effect of HSP at 0–100 μM/200 mg/kg concentration for 24 h and found that HSP induced apoptosis and prevented liver fibrosis. HSP decreased the level of AST, ALT, hydroxyproline (Hyp), HA, LN, TNF-α, IL-6, extracellular matrix (ECM) formation, Smad2/3 phosphorylation, and suppressed TGF-β1/Smad pathway [84]. Recently, researchers examined the impact of HSP derivatives on liver cancer both *in vivo* and *in vitro.* They found that HSP derivative decreased the level of ALP, ALT, AST, TGF-β1, HA, Hyp, F4/80+ macrophage infiltration, MCP-1, TNF-α, IL-1β, IL-6, TNF-α, and IL-1β, Gli-1, Shh expression at 25–100 mg/kg concentration in Littermate male C57BL/6J mice [85]. Additionally, HSP also repressed the phosphorylation and activation of NF-κβ-P65, preventing liver injury and hepatic fibrosis in the mice [85]. On the other hand, at 1–4 μM doses, HSP diminished α-SMA, Col1α1, Col3α1, TIMP-1, PAI-1, Gli-1 expression and increased the level of Bax and Caspase-3 in LX-2 hepatic cell, resulting in apoptosis [85]. HSP (20 mg/kg b.wt) promoted cell damage and mitigates liver enlargement in male Wistar albino rats [86]. A substantial increase in the level of SOD, CAT, GPx, GR, and GSH was observed in the albino rats when treated with HSP. In addition, nodules number, lipid peroxides, hydroperoxides, AST, ALT, ALP, LDH, and γGT were reduced in HSP treated albino mice [86]. Zhang *et al.* experimented via mitochondrial pathway *in vitro* on HepG-2, SMMC-7721 and Huh-7 cell line and *in vivo* Xenograft mouse model and stated that HSP (25–600 μM) inhibited hepatocellular carcinoma by inducing apoptosis and cell viability. Besides this, HSP upregulated some intracellular ROS, ATP, Ca2+ and cytosolic components i,e AIF, Apaf-1, Cyt C, caspase-3, caspase-9, Bax, and down-regulated Bcl-2, mitochondrial AIF, mitochondrial Apaf-1, and mitochondrial Cyt C that mediate apoptosis of cancer cell [77].

In conclusion, HSP can provide anti-liver cancer activities by regulating oxidative stress via antioxidant-related enzymes activities and inducing apoptosis by activating the death receptor and its consequential mechanism. Furthermore, this phytoestrogen regulates cancer-related growth factors and receptors, suppressing some signal transduction pathways and inflammation.

### 7.6 Pancreatic cancer

Pancreatic cancer is a fatal disease since it is usually detected at an advanced stage, with a low survival rate [87]. Smokers and diabetics are at a higher risk of pancreatic cancer, although there is some evidence that chronic cirrhosis, high-fat diets, and earlier cholecystectomy are linked to a greater risk of developing pancreatic cancer. Apart from this, around 5–10% of pancreatic cancer occurs genetically [88]. Therefore, effective treatment is required to prevent pancreatic cancer, and HSP has been beneficial in treating pancreatic cancer in recent years.To find the impact of HSP on the pancreatic cancer cell, J. Lee and his colleagues conducted a study on Miapaca-2, Panc-1, and SNU-213 cell lines at various doges, ranging from and found that HSP(0–20 μM) inhibited the migration of the treated cell [10]. Moreover, HSP treatment substantially reduced cell viability in Panc-1 pancreatic cancer cells at 2.5 μM concentration [10]. HSP obstructed the intracellular signalling, i.e., focal adhesion kinase (FAK), p38 phosphorylation, with activating caspase-3, leading to induction of apoptosis [10]. Researchers also established a Panc-1 xenograft model in BALB/c nude mice at 30 mg/kg concentration to evaluate the impact of HSP and found that it shows an anti-growth effect by activating Caspase-3 [10]. Patil *et al.* proved that dietary HSP stimulated apoptosis in human Panc-28 pancreatic cancer cells through increasing Bax, caspase-3 with concomitant decreasing Bcl-2 protein level and significantly increasing tumour suppressor protein p53 [89].

So HSP can prevent pancreatic cancer progression by inducing apoptosis by several molecular pathways.

### 7.7 Kidney cancer

Renal cell carcinoma (RCC) and renal transitional cell carcinoma (RTCT) arise from the renal parenchyma and renal pelvis and are the most prevalent types of kidney cancer in adults. It affects adults more than children [90]. Nowadays, due to the ability to target multiple pathways, HSP possesses the potentiality to treat cancer in the kidney. According to Chen *et al.* HSP caused apoptosis and attenuated nephrotoxicity in HK2 cell and AKI mice for 2.5–10 μM/50 mg/kg doses after 24 h treatment. HSP increased Nrf2 signalling, SIRT6, NQO1, HO-1 expression, down-regulated SCr, blood urea nitrogen (BUN), MDA, MPO, GSH, SOD, NOX4 expression level, thereby relieving cisplatin-induced acute kidney injury [14]. HSP down-regulated the level of MDA, TNF-α, IL-1β, IL-6, lipid peroxidation, oxidative stress; Hence reduced cisplatin-induced nephrotoxicity in male, Wister rate at 50–100 mg/kg [91]. Wang and his co-workers performed a study both *in vivo* and *in vitro* on (NRK)-52E cell line and UUO-mouse model to evaluate the efficacy of HSP at 50–100 μM and 60 mg/kg/day concentration, respectively. Their study proved that HSP decreased the expression of fibronectin (FN), Collagen I, α-SMA, EMT, Shh, Gli-1, deceased E-cadherin expression, reducing renal fibrosis normalize the renal function [92].

To sum up, cancer treatment in the kidney, HSP activate gene associated with antioxidant enzyme, regulates signalling mechanism, metastasis, and some inflammatory biomarkers.

### 7.8 Gastric cancer

Gastric cancer, also known as stomach cancer, is one of the deadliest and complex diseases, which has two main sites, i.e., proximal and distal, with a higher incidence in men [93, 94]. Gastric cancer is caused by various factors, including the environment, food and lifestyle, genetics, pre-malignant stomach lesions, socioeconomic position, *Helicobacter pylori*, Epstein Barr virus, and smoking [95]. Researchers performed several investigations and confirmed that HSP is an effective natural compound for the treatment of gastric cancer. HSP-treated gastric cancer inhibited cell proliferation by inducing apoptosis by increasing Bax/Bcl-2 ratio, cyt-c,caspase-3, caspase-9, AIF, and Apaf-1 via a mitochondrial-dependent pathway *in vitro* and xenograft tumours at a dose-dependent manner [96]. Similarly, Bagheri et al. reported that HSP (200–400 mM) reduces the ROS level of GCSCs [97]. Recently, Wang et al. (2021) stated that the treatment of HSP in gastric cancer cells decreased cell migration and invasion by suppressing genes expression related to the metastatic and reducing disruptor of telemetric silencing 1-like (DOT1L) and the methylation of histone H3K79 in a gastric cancer cell by regulating the activity of CBP [98]. Moreover, Hesperidin (100 μM) could change cell morphology i,e, chromatin condensation, and apoptosis markers like alter Bcl-2, caspase-3 activation in SNU-668 human gastric cells possible usage of hesperidin in gastric cancer patients [99].

Thus, HSP has the possibility as a potential therapeutic agent for gastric cancer through regulation of oxidative stress and mitochondrial-dependent apoptosis pathway.

### 7.9 Oral cancer

The development of HSP-loaded Eudragit-E nanoparticles (HETNPs) showed anti-cancer potency in oral carcinoma (KB) cells. High levels of ROS value, loss of mitochondrial membrane potential (MMP), and apoptotic morphological changes are more effectively shown with HETNPs than native HSP [17]. 7,12-Dimethylbenz[a]anthracene (DMBA) induced oral carcinoma showed reduction at the emission of collagen, nicotinamide adenine dinucleotide (NAD), and flavin adenine dinucleotide (FAD) but with the oral administration with HSP and its nanoparticles restore the endogenous fluorophores emission and higher redox ratio in the buccal mucosa of DMBA animal [100]. Oral tumour development in buccal pouches by DMBA causes abnormalities such as hyperplasia, dysplasia, and higher cell proliferation in squamous cell carcinoma (SCC). Treatment with HSP attributed anticancer effect through mediating apoptotic and anti-proliferative properties by decreasing the above expression [26]. Inhibition of angiogenesis is considered one of the effective ways of blocking cancer growth. HSP (40 mg/kg body weight/day) reduces cell growth by downregulation of vascular endothelial growth factor (VGEF) in DMBA treated tissue with its nanoparticles. These findings prove that HSP, along with its nanoparticles, will use as a medicine in oral cancer in the future needs more attention [101].

### 7.10 Ovarian cancer

Like previous cancer in our review, HSP also possesses anticancer activities against ovarian cancer via numerous mechanisms. HSP attributes apoptosis in SK-OV-3 ovarian cancer cells through the upregulation of ROS [20]. However, hesperidin (aglycone form of HSP) inhibits the proliferation of (A2780) ovarian cancer cells and induces apoptosis by altering the endoplasmic reticulum stress signalling pathway [102]. Additionally, hesperidin showed cytotoxic to the ovarian cancer cell. These phytochemicals promote antioxidant activity and induce apoptosis through activating cleaved caspase-3 in ovarian cancer cells [103].

### 7.11 Glioblastoma

Glioblastoma (GBM) is the most frequent and severe malignant brain cancer in elderly adults, mainly affecting the brain, but it can also occur in the brain stem, cerebellum, and spinal cord [104]. It involves complicated signalling pathways that are difficult to treat, and in most cases, the tumour develops spontaneously, while in other cases, cancer grows through the malignant progression of a lower-grade brain tumour [105]. Our targeted phytochemicals have a positive effect on glioblastoma cancer. In U-251,-87 glioblastoma cells, HSP diminishes cell viability when administered at the dose of 100–800 μM. HSP induces apoptosis in a dose-dependent manner by decreasing Bcl-2 and increasing Bax protein expression and causes cell cycle arrest by reducing cyclin B1, CDK1 and increasing tumour suppressor gene p21 phosphorylation p38 MAPK, arresting the G2/M phase [12]. Ersoz *et al.* showed that in C6 glioma cell HSP at 0.1–200 μg/Ml doses increases cytotoxic activity and lowers cell viability. Furthermore, Immuno-cytochemical analysis revealed that HSP (25–100 μM) regulates apoptosis and cell proliferation through ROS generation and SOD enzyme activity [51]. *In vivo* analysis of Wister rat with implanted C6 glioma cells showed that HSP inhibited tumour growth when administered at 10–20 mg/kg. This study also revealed that HSP-activated caspase-3 and -9, increased Bax/Bcl-2 ratio; hence inducing apoptosis and down-regulated HIF-1α, VEGF, VEGFR2 signalling pathway, reduced the expression of cyclin B1 and D1 with upregulating Claudin-1, ZO-1 expression, leading to decreasing cancer cell proliferation [106].

In a nutshell, naturally occurring HSP possesses anti-Glioblastoma properties by regulating some proteins associated with apoptosis, cell cycle, glucose transportation, and growth factors.

### 7.12 Other types of cancer

Some others types of cancer are also treated by HSP**.** In leukemia HL60 cell lines, HSP (at 1–200 μM) displayed an anti-cancer effect by inducing apoptosis by increasing MMP loss, caspase-3 activity, and arresting the cell cycle at G2/M and G0/G1 phase [22]. Furthermore, SAM and SH3 domain protein -1, metallothionein 1F (MT1F) and 1A (MT1A), small proline-rich protein 2D (SPRR2D), and 2F (SPRR2F), TXNIP, and MAP3K1 gene expression were increased at 100–150 μM HSP concentration. HSP also down-regulated tubulin beta-1 chain (TUBB1), TUBA1C, TUBB2C, ID1, ID3, neuromedia U, neuromedin U (NMU), FGFR-3, calcium-binding protein –P, ribosomal protein S25 (RPS25), and C-MYC gene expression at 100–150 μM HSP doses [22]. Another study by the same researchers was performed on a different leukemia cancer cell line, i.e., K562, and found that HSP promotes apoptosis in K562 leukemia cells also at the same dose. In this leukemia cell, HSP arrested G0/G1 phase and enhanced DUSP1 (dual specificity phosphatase 1), DUSP3, DUSP5, CDK1A, CDK1B GADD45B, SPRR2D, MT1F, MT1A, p27Kip1, CASP4, NFKBIA gene expression at 100–150 μM concentration [107]. Patel *et al.* reported that HSP promotes apoptosis and reduces cell proliferation in the HTh7 thyroid cancer cell line at 0–400 μM doses. They also found that HSP upregulated PARP cleavage, Notch 1 signalling, caspase-3, luciferase activity, and BAD expression [21]. However, some transcriptor in thyroid i,e TTF1, TTF2, PAX8, thyroid-stimulating hormone receptor (TSHR), sodium/iodide symporter (NIS) were increased, and the level of survivin was decreased after 48–72 h HSP (0–400 μM) treatment [21]. In a study conducted by Zarebczan *et al.*, HSP showed the potential effect to treat carcinoid cancer on BON cells at 0–100 μmol/L concentration. HSP prevented BON cancer cell proliferation and induced cancer cell death by decreasing achaete-scute complex-like 1(ASCL1), chromogranin A (CgA), and enhanced Notch 1 expression [108]. At 0–1000 μM concentration, HSP induced apoptosis and reduced cell proliferation and viability after 24–48 h treatment in SiHa cervical cancer cells. In addition, HSP mitigated mitochondrial membrane potential with increasing caspase-9,-8,-3, p53, Bax, Fas, FADD (Fas-associated death domain-containing protein) expression, leading to arrest cell cycle at G2/M phase [109]. An *in vitro* analysis on A431 human carcinoma cells revealed that HSP (10–500 μM) upregulated ROS production, JNK1/2, p38, Bax, p21 expression and suppressed the expression of ERK1/2, cyclin B1, D1, D3, E1, thereby leading to apoptosis and reduced cell viability [110]. Lentini *et al.* performed research on murine B16-f10 melanoma cell and male C57BL/6 mice *in vitro* and *in vivo* at 10 μM concentration for 24–72 h treatment. The researchers found that HSP suppressed the level of polyamine, spermidine, spermine and upregulated the activity of transglutaminase (TGase), thereby inhibiting cell growth and metastasis [111]. Furthermore, a recent study on the same cell line and female C57BL/6 mice demonstrated that HSP at 125 mg/mL doses could inhibit melanogenic tumour growth by activating PI3K-Akt signalling and cytotoxic T lymphocyte and suppressed the tolerogenic T cell response [112]. HSP induced apoptosis in both *in vitro* (Eca-109) and *in vivo* (female BALB/C nude mice) on oesophagal Eca-109 cancer cells at 0–500 μM/30–90 mg/kg concentration. Moreover, HSP increased ROS production, cyt-c, caspase-9,-3, Apaf-1, Bax, Sufu (suppressor of fused) expression and decreased GSH, Bcl-2, and survivin expression [18]. A recent study on the Eca-109 esophageal cell line revealed that HSP when administered at 100–300 μM concentration, suppressed cell proliferation and invasion after 24 h treatment. Additionally, reduction in PI3K/AKT signalling pathway, cyclin D1, MMP-2,9, PI3K-p85 expression, and elevation in PTEN phosphorylation and p21 expression were noticed in HSP treated Eca-109 cell line, which causes cell cycle arrest at G0/G1 phase [113]. Overview of HSP anticancer effect against numerous types of cancer is summarized in Table 1.

**Table 1:** Overview of HSP anticancer effect against numerous types of cancer.

Cancer type	Dose	Type of study (*In vitro and in vivo*)	Molecular mechanism	Molecular target	Reference
Breast cancer	100 μM	*In vitro* MCF-7	↑Apoptosis ↓Mammosphere ↓Colony formation ↓Migration	↑Arrest G0/G1 phase ↑p53, PPARG, NOTCH1 ↓β-catenin	[56]
	1–100 μM	*In vitro* MCF-7	↓Cell proliferation ↑Apoptosis	↑Arrest G1 phase ↓CDK2, CDK4, Cyclin D, Cyclin E ↑p21Cip1 and p27Kip1 ↑CDK4-p21Cip1 ↑Bax ↓Bcl-2	[57]
	20–200 μM	*In vitro* MCF-7,MCF-10A, HMEC, MDA-MB-231	↑Apoptosis ↓Cell proliferation ↑DNA damage	↑Caspase 3,7,9 ↑PARP, Bax:Bcl2, ROS, JNK, SK1 ↑ASK1/JNK pathway, ↑Cytochrome c	[62]
	1–20 μM	*In vitro* MCF-7	↓Cell proliferation	↓Blocked Ahr ↓CYP1A1, CYP1A2, CYP1B1	[58]
	5–100 μM	*In vitro* MDA-MB-231	↓Cell proliferation	↓GLUT1,GLUT4,IR-beta phosphorylation, Akt	[63]
	500–5000 ppm	*In vitro and* MCF-7 *In vivo* Female athymic mice	↓Cell proliferation	↓Aromatase enzyme ↓Cyclin D1,CDK4,Bcl-xL, pS2 ↑p57Kip2 expression	[59]
	10–500 μM	*In vitro* SKBR3, MDA-MB-231	↑Apoptosis ↓Cell growth	↓HER2-TK activity, ↓MMP loss ↑Chromatin condensation ↑Caspase 3 ↑Arrest G2 phase	[65]
	N/A	*In vitro* MDA-MB-231	↓Cellular viability, metastatic potential	↑miR-486-5p	[64]
	95 μM	*In vitro* MCF-7/HER2, MCF-7/EV	↓Cell viability, invasion, migration ↑Apoptosis	↑Arrest G2/M phase ↓HER2,MMP-9, Rac1, lamellipodia	[61]
	0–20 μM	*In vitro* MCF-7	↓Tumor growth	↓Aromatase,↑CCAAT/C/EBP,pERK-1&-2	[60]
	50–100 μM	*In vitro* 4T1	↑Apoptosis, ↓Metastasis	↑SubG1 ↓MMP-9	[66]
Lung cancer		*In vitro* H441 cell	↑ Apoptotic cell death ↓ Cell viability ↓Cell migration ↓Angiogenesis	↓TGF-β ↓Glucose transporters	[67]
	16 μM	*In vitro* A 549 cell	↑Apoptosis	N/A	[49]
	100 μM	*In vitro* A549 cell	↓Cell viability ↓Cell proliferation	↓IL-1β, CDK ↑p21, p27 ↓COX-2, MAPK, ERK1/2, NF-κBp65 expression ↓PGE2 expression	[70]
	58–1000 μM 40 mg/kg	*In vitro* A549 cell *In vivo* C57BL/6	↓Cell proliferation ↓Migration ↓Cell growth	↓ki67 expression ↑Cell cycle arrest ↑ROS	[117]
	50 mg/kg	*In vivo* Swiss albino mice	↓Angiogenesis ↑Apoptosis	↓NF-κB, PCNA, CYP1A1 ↓TNF-α, Nrf2, GR, GST	[71]
Prostate	0–100 μM	*In vitro* (PC-3)	↓Cell proliferation ↑Apoptosis	↓Bcl-xL, NF-κβ ↑BAD, Bax	[74]
	0–700 μM	*In vitro* PC-3	↓Cell proliferation ↓Cell viability	↑pSTAT3, pERK1/2, pAKT signaling ↑IL-6 gene expression ↑Arrest G0/G1 phase	[75]
	0–1000 μM	*In vitro* PC-3	↑Apoptosis ↓Cell proliferation	N/A	[11]
	12.5–200 μM	*In vitro* PPC-1	↑Apoptosis ↓Cell viability	↑Arrest G1/S phase ↑Mitochondrial membrane depolarization	[53]
Colon cancer		*In vivo* Balb/c-nu/nu nude mice	↓ Cell proliferation ↑ Apoptosis	↑ ROS, ca^2+^and ATP, BCL-2 ↑AIF, Apaf-1, Cyt C, caspase-3,9, Bax	[77]
	20 mg/kg body weight/day	*In vivo* DMH induced rat colon carcinogenesis	↑Apoptosis ↓Angiogenesis	↓VGEF, EGE, Bfgf ↓BCL-2, COX-2 ↑Bax level	[15]
	20 mg/kg body weight/day	*In vivo* 1,2-DMH induced rat colon carcinogenesis	↓Tumor multiplicity ↑Apoptosis	↑Antioxidant enzymes including SOD, CAT, GPX, GR, and GSH levels	[78]
	10,20,30 mg/kg body weight/day	*In vivo* DMH-induced colon carcinogenesis in rat	↓Tumor multiplicity ↓Tumor size ↓ Cell growth	↓Lipid peroxide ↑Antioxidant enzyme activity	[79]
	5–100 μM	*In vitro* HT-29 cell line	↓Cell growth ↑Apoptosis	↑Cytochrome-c, Bax, caspase-3 ↓BCL-2 ↑SOD, CAT, GPx	[81]
	20 mg/kg body weight/day	*In vivo* DMH-induced colon carcinogenesis in rat	↓Cell proliferation	↓ PCNA index, proliferation marker ↓ACF, formation of foci	[142]
	0–100 μM	*In vitro* HCT-116 cell line	↓Cell viability ↑Apoptosis	↑JNK-1, JNK-2 ↑Caspase9, caspase3	[80]
	0–80 μM	*In vitro* HCT-15	↓Cell growth ↑Apoptotic cell death	↑ Folate receptor expression ↑Bax and Bad mRNA	[50]
	15 mg/kg	*In vivo* male Wistar rats	↑Apoptosis	↑ SOD2,CAT, GR ↓ GPx	[83]
Liver cancer	50 mg/kg/day	*In vivo* Sprague-Dawley rat	↑Apoptosis	↑ Fas and FasL, Albumin, Caspase-8,3 ↓AFP, Bcl-2, GSH, ALT, AST, Bilirubin	[13]
	0–100 μM/200 mg/kg	*In vitro* HSC-T6 *in vivo* male C57 mice	↑Apoptosis, ↓Liver fibrosis	↓ TNF-α, IL-6, ALT,AST, LN, HA, Hyp, ECM, TGF-β1/Smad pathway, pSmad2/3	[84]
	25–100 mg/kg/1–4 μM	*In vitro* LX-2 *In vivo* Littermate male C57bL/6J mice	↑Apoptosis ↓Hepatic fibrosis, Liver injury	↓ALT,ASP,ALP,TGF-β1,HA,Hyp,α-SMA,Col1α1,Col3α1,TIMP-1,F4/80+ macrophage infiltration,MCP-1,TNF-α,IL-1β,IL-6,NF-κβ-P65 phosphorylation,PAI-1,Gli-1,Shh ↑Bax, Caspase-3	[85]
	20 mg/kg b.wt	*In vivo* male wistar albino rats	↑Cell damage, ↓Liver enlargement	↑SOD,CAT, GPx, GR,GSH ↓Nodules number, lipid peroxides and hydroperoxides, AST, ALT, ALP, LDH, γGT	[86]
Pancreatic Cancer	0–20μM/30 mg/kg	*In vitro* Miapaca-2, Panc-1,SNU-213 *in vivo* BALB/c nude mice	↓Cell growth, ↓Cell viability ↓Migration	↑Caspase-3 ↓Fak,p38 signaling, intracellular signaling	[10]
	N/A	*In vitro* Panc-28	↑Apoptosis	↑Bax,↓Bcl-2,↑Bax/Bcl2 ↑Caspase-3,p53	[89]
Kidney cancer	2.5–10 μM/50 mg/kg	*In vitro* HK2 *In vivo* AKI mice	↑Apoptosis, ↓Nephrotoxicity	↑Nrf2 signaling, SIRT6, NQO1, HO-1 ↓SCr, BUN, MDA, MPO, GSH, SOD, NOX4	[14]
	50–100 mg/kg	*In vivo* male Wistar rat	↓Nephrotoxicity ↓Lipid peroxidation	↓MDA, TNF-α, IL-1β, IL-6,	[91]
	100–300 μM	*In vitro* Eca-109 cell line	↓Cell proliferation ↓Cell invasion	↑Arrest G0/G1 phase ↑PTEN phosphorylation, p21 ↓PI3K/AKT, cyclin D1, MMP-2,-9, PI3K-p85 expression	[113]
Oral cancer	40 mg/kg body	*In vivo* Hamster buccal pouch	↑Apoptosis ↓Cell growth	↓VEGF level ↓PpIX levels	[101]
		*In vivo* Hamster buccal pouch	↑Apoptotis ↓Cell proliferation	↓Mutant-p53, Caspase-3 and caspase-9 and cyclin-D1, β actin ↓DMBA induced oral cancer	[26]
Ovarian cancer	245 μM	*In vitro* SK-0V-3	↑Apoptosis		[20]
Glioblastoma	100–800 μM	*In vitro* U-251, U-87	↓Cell viability ↑Apoptosis	↑Bax,/Bcl-2 ↑Arrest G2/M phase ↓Cyclin B1,CDK1 ↑p21, p38 MAPK	[12]
	0.1–200 μg/mL	*In vitro* C6	↓Cell proliferation ↓Cell viability ↑Apoptosis	↑ROS generation, SOD enzyme ↑Cytotoxic activity	[51]
	10–20 mg/kg	*In vivo* Wistar rat with C6 glioma cells	↑Apoptosis ↓Cell proliferation	↑Caspase-9,-3, ↓ Bax/Bcl2 ↓Cyclin B1,D1 ↓HIF-1α,VEGF, VEGFR2 ↑Claudin-1, ZO-1 expression	[106]

↑ = Increase; ↓ = Decrease.

## 8 Potential synergy of HSP with other agents in cancer treatment: mechanistic insight

Cancer is a devastating disease globally, and natural products have already proven significant therapeutic value in cancer treatment [114]. Combining these natural products showed a remarkable result against cancer and reduced many drugs' side effects. Recent studies of HSP revealed that this phytochemical is widely used with other natural agents. In the panc-1 cell line, HSP was evaluated against pancreatic cancer cells, but the compounds had low activity. However, when HSP was used in conjunction with naringin and naringenin, the phosphorylation of FAK and the p38 signalling pathway was downregulated, which was not the same with either of the mother therapies [10, 115]. Similarly, one study showed that naringenin had few activities in HER2, but the combination of HSP and naringenin has been strongly associated with inhibiting tyrosine kinase activation of the HER2 receptor protein [65]. In another study conducted to evaluate combination therapy in breast cancer, luteolin-induced cell death in breast cancer with unknown mechanism, but in a combination of hesperidin increased apoptosis in MCF-7 cells [116]. Among some common traditional therapeutic approaches, still, platinum-based chemotherapeutics is widely used in non-small-cell carcinoma (NSCLC). Wang *et al.* reported that co-treatment of HSP with platinum attributes apoptosis-related cell death through downregulation of UGT1A3 with concomitant increasing ROS in a xenograft model [117]. Moreover, HSP is also used with chemotherapeutics drugs for better therapeutics advantage. Dextran with a combination of HSP improved antioxidant activity of HSP and induced cytotoxic effect on both MCF and AGS more than treatment with HSP alone [118]. Furthermore, the combination of HSP and Doxorubicin arrested the cell cycle at G2/M and mediated anti-metastasis activities via the downregulation of MMP-9 expression in 4T1 cells [66]. In addition, further study summarized that the combination of HSP with quercetin effectively induces antiproliferative effects in MDA-MB-435 cells because of its hydroxyl group at 3,4 position than HSP combined with genistein, galangin [119]. Again in esophageal cancer, Eca-109 cell, HSP combined with 5-FU (fluorouracil) inhibited cell growth through the downregulation of Bcl-2 and increased cleaved caspase-3, caspase-9 more effectively than did either drug alone [113]. Moreover, the inhibitory effects of HSP and etoposide on cell proliferation were cumulative. HSP also caused G2 phase arrest, which was linked to lower gene expression of cyclin B1 and E1 as well as cyclin-dependent kinases 1 and 2 in osteosarcoma U2OS cells [19]. Summary of the combined effect of HSP with other phytochemical and chemotherapeutics agents are shortlisted in Table 2.

**Table 2:** Summary of the combined effect of HSP with other phytochemicals and chemotherapeutic agents.

Cancer type	Combined agents	Study model	Combined target	Ref
Pancreatic cancer	Naringin	Panc-1	↓Phosphorylation of FAK and p38 signaling pathway	[115]
	Naringenin	Panc-1	↑Caspase-3 ↓ FAK and p38 pathway ↓ Cell growth	[10]
Breast cancer	Luteolin	MCF-7	↓ Anti-apoptotic, BCL-2, miR21 ↑Pro-apoptotic, Bax ↑miR26, -34a	[116]
	Dextran	MCF-7	↑Cytotoxic effect on cancer cell	[118]
	Doxorubicin	4T1	↑G2/M phase ↓MMP-9, Migration	[66]
	Naringenin	MDA-MB-231 SK-BR-3	↓HER2-TK activity ↓MMP	[65]
	Quercetin Genistein Galangin	MDA-MB-435	↓Proliferation ↑Apoptosis ↓Cell growth	[119]
	Letrozole	MCF-7	NA	[143]
Bladder cancer	Diosmin and hesperidin		↓AgNOR, BUdR ↑ Apoptosis	[144]
Esophageal cancer	5-FU	Eca-109	↓Bcl-2, P13K/AKT ↑Bax, caspase-3,caspase 9	[113]
Osteosarcoma	Etoposide	U2OS	↑G1 arrest, apoptosis ↓Cyclin B1, E1 ↓CDK1, CDK2	[19]
Gastric adenocarcinoma	Dextran	AGS	↑ROS ↓Cell viability and proliferation	[118]
Kidney cancer	Cisplatin	HK-2	↓Activation of Nrf2 ↓p-JNK, p-ERK, p38	[14]
Prostate cancer	Taxane (doxorubicin)	PPC-1	↓NF-ĸB, ↑p38 caspase-3	[132]
Gastric cancer	Cisplatin (DPP)	HGC-27, SGC-7901, MGC-803	↑PTEN, Cyt C ↓p-AKT, Cyclin D1	[145]

↑ = Increase; ↓ = Decrease.

## 9 Power of HSP in alleviating multidrug resistance in numerous cancer types

Treatment of cancer patients is becoming challenging due to major difficulties i,e multidrug resistance (MDR) [120]. This resistance mechanism can originate from several potential defensive mechanisms like drug efflux [121], drug inactivation [122], drug detoxification [122], drug target modification [123], involvement of cancer stem cell [124], miRNA dysregulation [125], epigenetic alteration [126] and other multiple mechanisms irregular DNA damage/repair mechanism, tumour microenvironment, modulating ROS [124, 126]. Several proteins are associated with occurring drug resistance i,e P glycoprotein(P-GP), MRP 1, MRP1–9, BCRP, and alteration in beta-tubulin [127]. Literature review suggests that several agents have been designed and used to overcome multidrug resistance. But the majority of these failed for the final destination due to having several side effects and less efficacy. Phytochemicals like HSP are available in citrus fruit can be used as a complementary therapy to multidrug resistance. Excess use of doxorubicin causes drug resistance by overexpressing P-glycoprotein (P-gp) via increasing drug efflux. But, HSP with doxorubicin treatment suppresses P-gp expression in MCF-7 and MCF-7/DOX by lowering the optimum concentration of both HSP and doxorubicin [128]. Furthermore, HSP might have the prospective to be developed as a co-chemotherapeutic agent combination with doxorubicin resulted in arresting cell cycle, induce apoptosis, suppressed Rac1, HER2, MMP9 expression, and cell migration in HER2 overexpressing breast cancer cells respective doses of 95 μM and 0.2 μM, where dose were 377 and 0.8 μM, respectively during individual testing [61]. Inhibiting NF-κB and IGF1R expression improves some anticancer drugs sensitivity in resistant cell lines [129, 130]. P-gp mediated MDR was reversed when HSP has treated A549/DDP cells by decreasing P-gp expression, which was directly linked with inhibition of transcription factor NF-κB signalling pathway [131]. The use of cisplatin in cancer treatment also causes nephrotoxicity. But HSP dramatically reduced apoptosis in the nephron by reducing ROS levels, activating the Nrf2 signalling pathway, and MAPK signalling pathway against inflammation in cisplatin-treated HK-2 cells and AKI mice [14]. Taxanes are a common group of drugs in prostate cancer treatment. But the use of these drugs causes toxicity to another normal cell. Sak et al. summarised that exposure of HSP with a combination of taxanes decreased cytotoxicity 9.8- and 13.1-fold for docetaxel and cabazitaxel, respectively, in the PPC-1 prostate cancer cell line [132]. HSP combined with 5-fluorouracil (5-FU) was more effective rather than a single use of the drug in Eca-109 cell line and a xenograft mouse model of esophageal cancer through down-regulating Bcl-2 with simultaneously increasing Bax, caspase-3, caspase-9 more effectively than did 5-FU only and reduced cancer cell invasion with cell proliferation through mitigating PI3K/AKT signalling pathway [113].

## 10 Comparative anticancer activities analysis

Numerous first-line anticancer drugs are recommended by the national cancer institute, NIH, for individual cancer. Our targeted phytochemical name HSP possesses a wide range of activities like standard drugs. It works either based on single or multiple anticancer pathways. Based on Table 3, it can be summaries that HSP may be one of the potent candidates for multiple cancers.

**Table 3:** Comparative anti-cancer activities of first line-medicines and Hesperetin for multiple cancer treatment.

Cancer	Standard drug and Hesperetin	Apoptosis induction	Cell proliferation inhibition	Metastasis inhibition	Angiogenesis inhibition
Breast cancer	Toremifene	Yes [146]			
	Hesperetin	Yes	Yes	Yes	Yes
Lung cancer	Alectinib	Yes [147]			
	Hesperetin	Yes	Yes	Yes	Yes
Prostate cancer	Docetaxel	Yes [148]			
	Hesperetin	Yes	Yes	NA	NA
Colon cancer	Capecitabine	Yes [149]			
	Hesperetin	Yes	Yes	NA	Yes
Liver cancer	Atezolizumab	Yes [150]			
	Hesperetin	YES	NA	NA	NA
Pancreatic cancer	Erlotinib	Yes [151]			
	Hesperetin	Yes	Yes	Yes	NA
Kidney cancer	Axitinib	Yes [152]			
	Hesperetin	Yes	Yes	Yes	NA
Oral cancer	Docetaxel	Yes [153]			
	Hesperetin	Yes	Yes	NA	NA
Ovarian cancer	Paclitaxel	Yes [154]			
	Hesperetin	Yes	NA	NA	NA
Glioblastoma	Temozolomide	Yes [155]			
	Hesperetin	Yes	Yes	NA	NA

## 11 Toxicity

Despite HSP attributing hundreds of therapeutics advantages, it may address some adverse effects or toxic reactions. HSP is directly associated with suppressing Der p 1 mediated HLA-DR, CD83, and CD86, expression in Dendritic cells. Furthermore, they found that HSP inhibited the Der p 1-induced IκB phosphorylation with inhibition of NF-κB p65 translocation [133]. Rajasekar et al. summarized that HSP treatment causes edema in the yolk-sac and pericardial, decreased heartbeat rate, upcurved tail, cardia chamber bulging, and curved body axis in the Zebrafish model [134]. Administration of HSP does not cause any allergic reaction, rather than it showed anti-allergic activity through suppressing histamine release from RBL-2H3 cells and suppressing prostaglandin E2 production with inhibiting cyclooxygenase-2 enzyme activities in RAW 264.7 cells cell lines [135]. In contrast to adverse effects in the brain, HSP may protect the brain from oxidative damage by activating the antioxidant enzyme system in mice's model system [136]. HSP could decrease triacylglycerol (TG) accumulation in the liver by reducing TG enzyme activity [137]. Furthermore, acyl-coenzyme A activity i,e gene associated with cholesterol acyltransferase (ACAT1 and ACAT2) is reduced or inhibited by HSP, and it decreases microsomal triglyceride transfer protein (MTP) activity, thereby lowering cholesterol levels [138]. But its mother source, hesperidin, attributed adverse effects in pregnant women [40], infertility, trauma, infection, and systemic diseases in an animal in vitro study [139, 140].

## 12 Pharmacokinetics and future perspective in drug development

Pharmacokinetic studies help determine the effects of respective drugs in the body based on absorption, distribution, metabolism, excretion, and toxicity. These pharmacokinetic properties are called ADMET [141]. Analysis of these properties aid in designing a drug to treat numerous diseases in medical society with understanding potential risk, time, and cost to determine whether or not a compound is suitable to proceed to the clinical stage. The pharmacokinetics profile of HSP was predicted using in-silico tools such as Schrodinger's QuickPro modules [23] and online accessible server admetSAR [24] and SwissADME [25] and tabulated in Table 4.

**Table 4:** Drug availability evaluation profile of HSP.

Pharmacokinetics parameter		Predicted remarks	Unit
Drug likeness	Lipinski	Yes	NA
	Jorgensen's	Yes	NA
	Ghose	Yes	NA
	Egan	Yes	NA
	Veber	Yes	NA
	Muegge	Yes	NA
	Bioavailability Score	0.55	NA
Absorption	Water solubility	1.803	Log mol/L
	Caco2 permeability	132.10	log Papp
	PercentHuman OralAbsorption	75.461	% Absorbed
	Skin Permeability	-4.071	log Kp
	P-glycoprotein substrate	Yes	Yes/No
Distribution	BBB permeability	-1.512	LogBB
	CNS permeability	-2	LogPS
	Human serum albumin	0.018	NA
Metabolism	CYP1A2 inhibitor	Yes	NA
	CYP2C19 inhibitor	No	NA
	CYP2C9 inhibitor	No	NA
	CYP2D6 inhibitor	No	NA
	CYP3A4 inhibitor	No	NA
Toxicity	Eye corrosion	No	NA
	Hepa-toxicity	Yes	NA
	AMES toxicity	No	NA
	hERG I inhibitors	No	NA
	Carcinogenicity	No	NA
	BSEP inhibitors	No	NA

It was observed that HSP was found to meet all tested i,e Lipinski, Jorgensen's, Ghose, Egan, Veber, and Muegge rules with a good bioavailability score (0.55), indicated maintained Drug likeness properties. The absorption of drugs is an essential property for entering blood circulation and practical activities [141]. Absorption features outlined that HSP is moderately soluble in water (QPlogPo/w = 1.803), moderate Caco-2 permeable, 75.45% absorbed human orally, act as a P-glycoprotein substrate, and have good skin penetrability (QPlogKp = -4.071). Distribution refers to drug movement within the body and depends on several factors i,e, BBB permeability, CNS permeability, binding to plasma protein, and many more [141]. Our predicted result suggested that HSP has the potentiality to cross the blood-brain barrier (QPlogBB = -1.512), penetrate to CNS (CNS = -2), and have a high affinity to bind with Human serum albumin (QPlogKhsa = 0.018). Human cytochrome P450 (CYP) isoforms found in the liver are associated with drug metabolism, and its inhibition causes drug toxicity in the body. The predicted metabolic result reported that HSP is the only inhibitor of CYP1A2 inhibitor rather than CYP2C19, CYP2C9, CYP2D6, and CYP3A4. Based on eye corrosion Hepa-toxicity, Carcinogenicity, AMES toxicity, hERG I inhibitors, and BSEP inhibitors, the toxicity profile of HSP was predicted. We found that HSP is toxic to liver, but non-toxic for other descriptors.

## 13 Concluding remark

**Figure 3:**
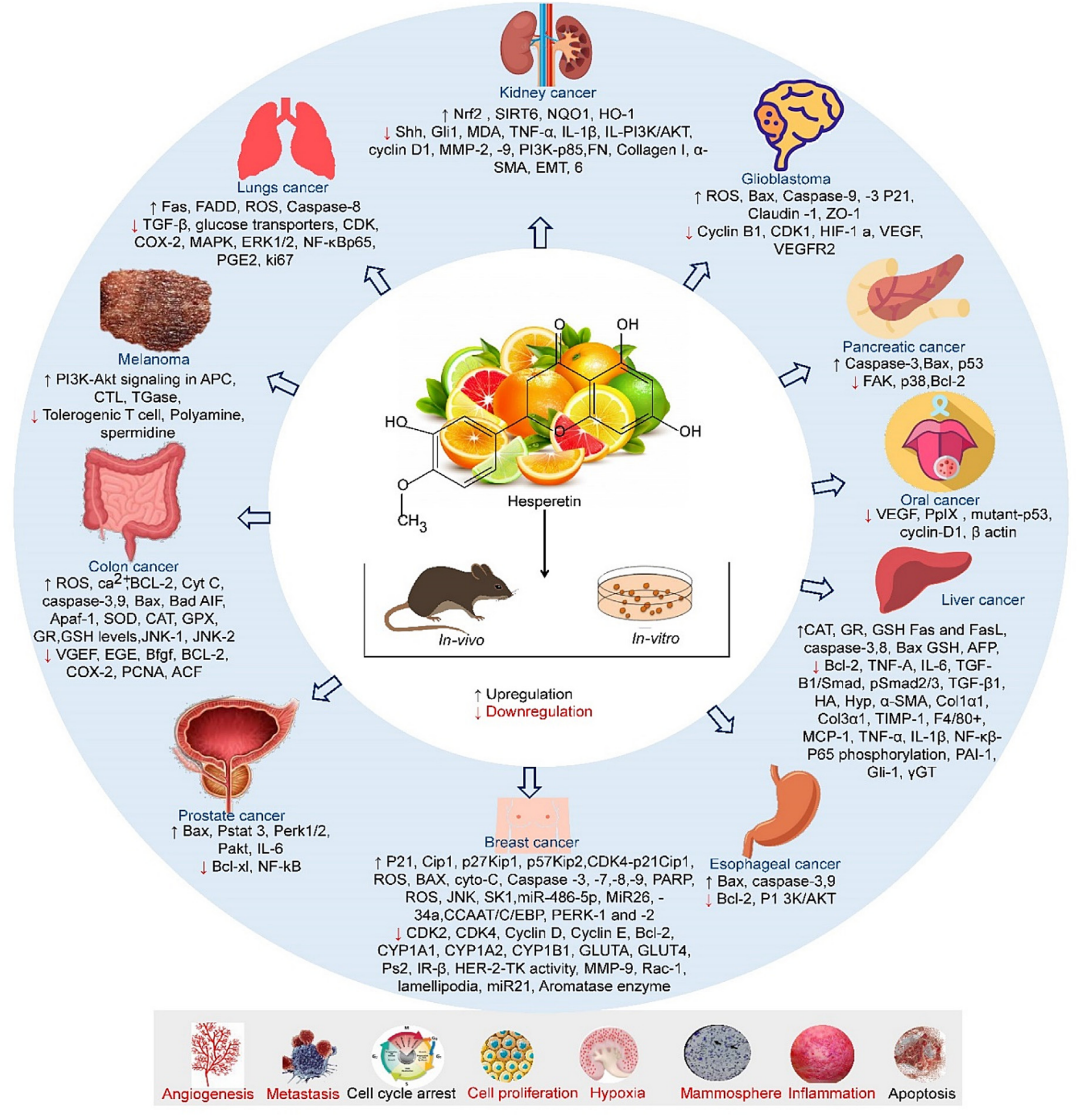
Molecular factors underlying anti-cancer activities of Hesperetin against numerous cancers.

HSP is a naturally occurring phytochemical primarily available in the fruit of citrus species, including oranges, grapefruit, and tangerines. The structural organization of HSP attributed its therapeutic advantages to humans against cancers. Using numerous cell lines of different cancer and animal model studies, it was found that HSP could lessen several carcinogenic events. The anticancer impact of HSP against several cancer developments is mediated through modulating some common receptors, growth factors, transcription factors, proteins, miRNA, enzymes, cytokines, cellular signalling pathways, and ROS production. This modulation regulates cell cycle arrest, cell proliferation, growth, viability, metastasis, angiogenesis, epigenetic factors, and apoptosis-related cell death in multiple cancer types(Figure 3). The combined effect of HSP with other phytochemical and chemotherapeutic agents increased the efficacy of other phytochemicals and chemotherapeutic agents with sensitizing, reducing side effects, and attenuating the resistance pattern of anticancer drugs in cancer patients. These high anticancer activities may be due to structural organization and higher bioavailability. Moreover, having better pharmacokinetics with lower toxicities and some *in silico* anticancer activities of HSP suggests that this phytochemical can be a potent candidate for drug discovery after performing the rest of the methods like molecular docking simulation for drug design against numerous diseases, especially cancer. For additional information about the anticancer activity, authors recommend long-term animal and clinical trial studies to understand better therapeutics advantages in cancer treatment and possible toxicities if they existed. So until the specific drugs are available in medical sectors, authors suggest HSP should be included as a dietary supplement to treat cancer patients.

## Declarations

### Author contribution statement

All authors listed have significantly contributed to the development and the writing of this article.

### Funding statement

This research did not receive any specific grant from funding agencies in the public, commercial, or not-for-profit sectors.

### Data availability statement

Data included in article/supplementary material/referenced in article.

### Declaration of interests statement

The authors declare no conflict of interest.

### Additional information

No additional information is available for this paper.
